# Accuracy of vascular tortuosity measures using computational modelling

**DOI:** 10.1038/s41598-022-04796-w

**Published:** 2022-01-17

**Authors:** Vishesh Kashyap, Ramtin Gharleghi, Darson D. Li, Lucy McGrath-Cadell, Robert M. Graham, Chris Ellis, Mark Webster, Susann Beier

**Affiliations:** 1 Mechanical and Aerospace Engineering Department, Henry Samueli School of Engineering and Applied Science, University of California, Los Angeles, USA; 2 School of Mechanical and Manufacturing Engineering, University of New South Wales, Sydney, NSW Australia; 3 Molecular Cardiology and Biophysics Division, Victor Chang Cardiac Research Institute, Sydney, NSW Australia; 4 Auckland Heart Group, Auckland, New Zealand; 5 Auckland City Hospital, Auckland, New Zealand

**Keywords:** Computational models, Statistical methods

## Abstract

Severe coronary tortuosity has previously been linked to low shear stresses at the luminal surface, yet this relationship is not fully understood. Several previous studies considered different tortuosity metrics when exploring its impact of on the wall shear stress (WSS), which has likely contributed to the ambiguous findings in the literature. Here, we aim to analyze different tortuosity metrics to determine a benchmark for the highest correlating metric with low time-averaged WSS (TAWSS). Using Computed Tomography Coronary Angiogram (CTCA) data from 127 patients without coronary artery disease, we applied all previously used tortuosity metrics to the left main coronary artery bifurcation, and to its left anterior descending and left circumflex branches, before modelling their TAWSS using computational fluid dynamics (CFD). The tortuosity measures included tortuosity index, average absolute-curvature, root-mean-squared (RMS) curvature, and average squared-derivative-curvature. Each tortuosity measure was then correlated with the percentage of vessel area that showed a < 0.4 Pa TAWSS, a threshold associated with altered endothelial cell cytoarchitecture and potentially higher disease risk. Our results showed a stronger correlation between curvature-based versus non-curvature-based tortuosity measures and low TAWSS, with the average-absolute-curvature showing the highest coefficient of determination across all left main branches (*p* < 0.001), followed by the average-squared-derivative-curvature (*p* = 0.001), and RMS-curvature (*p* = 0.002). The tortuosity index, the most widely used measure in literature, showed no significant correlation to low TAWSS (*p* = 0.86). We thus recommend the use of average-absolute-curvature as a tortuosity measure for future studies.

## Introduction

Mild coronary tortuosity, marked by larger vascular bending angles, is common and can even be found in people lacking coronary artery disease^1,2^. Strong tortuosity is a clinical anomaly, however, and defined by tight vessel curvature or bending^3^. Increased coronary tortuosity has been reported in patients suffering from spontaneous coronary artery dissection (SCAD)^4^, a condition with a high prevalence in females, and a global scoring system based on the number and angle of curves in the coronary arteries has been proposed for the use in evaluating tortuosity in these patients^4^. Increased coronary and other vascular tortuosity is also more common in the elderly^5^, and those with hypertension^6^. Tortuosity has also been associated with inherited arteriopathies, such as Loeys-Dietz Syndrome and Marfan Syndrome^5^. It is hypothesized that the mechanism linking severe coronary tortuosity to adverse clinical outcomes is that tortuosity-induced blood flow alterations result in non-physiological, adverse shear stress at the luminal vessel wall^7,8^. However, coronary tortuosity has been reported to be more common in women albeit, paradoxically, women have less severe coronary artery disease than men^9^. Together, the clinical relevance of tortuosity has yield inconsistent and contradictory results^10–12^.

Several studies have aimed to understand the link between blood flow and tortuosity in coronary arteries, whereby two different tortuosity metrics have been predominantly used: (i) the tortuosity index^2,3,13,14^, defined as the ratio between the length of a vessel segment and the distance between its start and endpoint, and (ii) the absolute-curvature measure^2,3,13,15–20^. These were studied in both idealized^3,14,15^ and patient-specific computational models^2,13,14,16^, and experimental work was also undertaken for idealized^18,19^ and patient-specific coronaries^20^.

Much of the available literature is focused on factors affecting Wall Shear Stress (WSS)^21^ or equivalently Endothelial Shear Stress (ESS). Low WSS values have been associated with higher disease risk, whereby in vivo studies in rabbit^22^ and swine^23^ have shown close association between areas of low WSS and endothelial cell proliferation leading to neointimal hyperplasia. Computational studies, which have used idealized models^3,24^, have reported that increased vessel tortuosity adversely effects flow resulting in higher flow helicity and overall lower WSS.

Patient-specific geometric considerations were found to dampen this effect overall^3^, which is likely due to small local changes in curvature on the arterial surface. For patient-specific studies, there appears to be a general disagreement between the specific effects of tortuosity on haemodynamics in the separate branch segments of the left main coronary. Specifically, when using the tortuosity index as a measure, there was a strong correlation between tortuosity and larger areas of low shear stress only in the left main coronary artery (LMCA) and left arterial descending (LAD) artery^2,16^. For tortuosity in the left circumflex (LCx) artery, one study showed a weak correlation^2^ with low Time Averaged WSS (TAWSS) whereas another showed a strong negative correlation^16^. Contrary to both these findings, another study found that the wall shear stress in LMCA decreased overall with tortuosity^3^. When absolute-curvature was used as the tortuosity measure, no correlation was found between vessel shape and TAWSS^2^, yet others reported a strong negative correlation between LCx artery curvature and TAWSS^16^.

**Figure 1:**
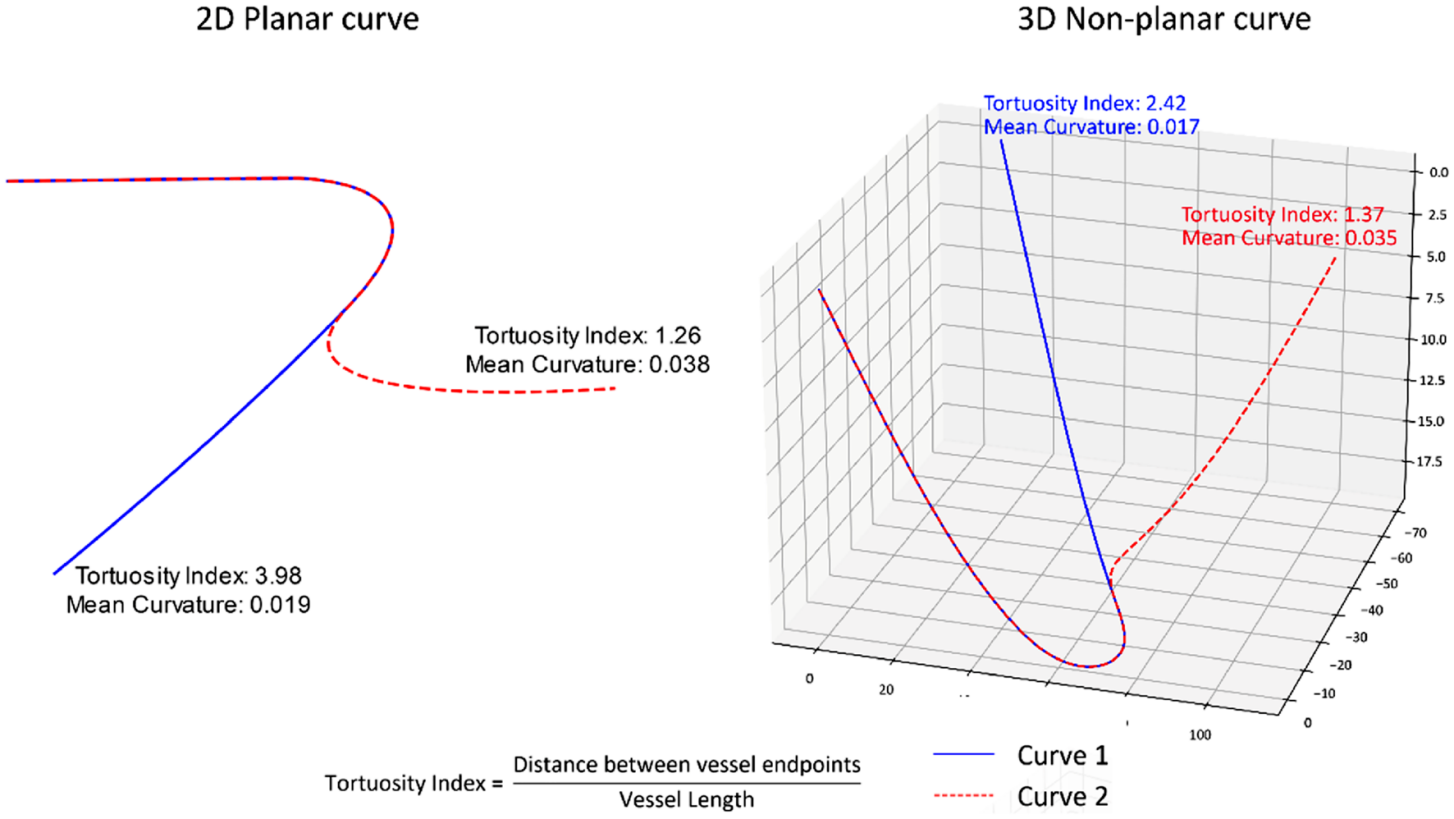
The behavior of mean curvature is more consistent and intuitive than the tortuosity index, in both 2- and 3-dimensional cases (left to right). Among the two lines shown, the one with an extra curved segment (red) has a greater mean curvature, but a significantly lower value of tortuosity index compared to the line with only a single bend (blue). This highlights an inherent issue with metrics such as tortuosity index, which do not consider the entire geometry.

It becomes apparent that there remains a lack of understanding of the effect of tortuosity and how to best measure its clinical impact. Tortuosity index is the most commonly used tortuosity metric due to its ease of calculation and clinical applicability, and thus is the most widely published measure in this context. However, Fig. 1 shows an example where the reliance on the tortuosity index can lead to aberrant results. In fact, following the mathematical definition of tortuosity in 3D space^25^ described as 3D curves, the definition of tortuosity index does not account for actual tortuosity in 3D space. However, the tortuosity index has been deemed a useful metric in clinical practice and research due to its ease of calculation and clinical applicability with 2D medical imaging, given that many important coronary imaging modalities (invasive CA and CTCA) utilize 2D representations of 3D structures. As a result, it is commonly considered as a clinically relevant measure and has been widely published as a measure for arterial tortuosity.

The main aim of this study is therefore to explore major tortuosity measures and their ability to predict low TAWSS (< 0.4 Pa) values that are non-physiological. To do so we consider the largest number of patient-specific left main coronary geometries to date (n = 127, n = 8 being the largest number considered previously^2,13^), but also include tortuosity measures which are commonly used in other vessels. Specifically, we analyzed (i) the tortuosity index commonly used in the carotid^26^, retinal^17^, cerebral^27^ and abdominal arteries^28^, (ii) the absolute-curvature used in carotid^29^, femoral^30^ and retinal vessels^31–33^, (iii) the squared-curvature used in retinal vessels^33,34^, and (iv) the square-derivative-curvature^35^. With this study design, we aim to make a meaningful recommendation for a tortuosity measure most suited for analysis of hemodynamics, and one that has important clinical implications.

## Methods

### Left main coronary artery geometries

**Figure 2:**
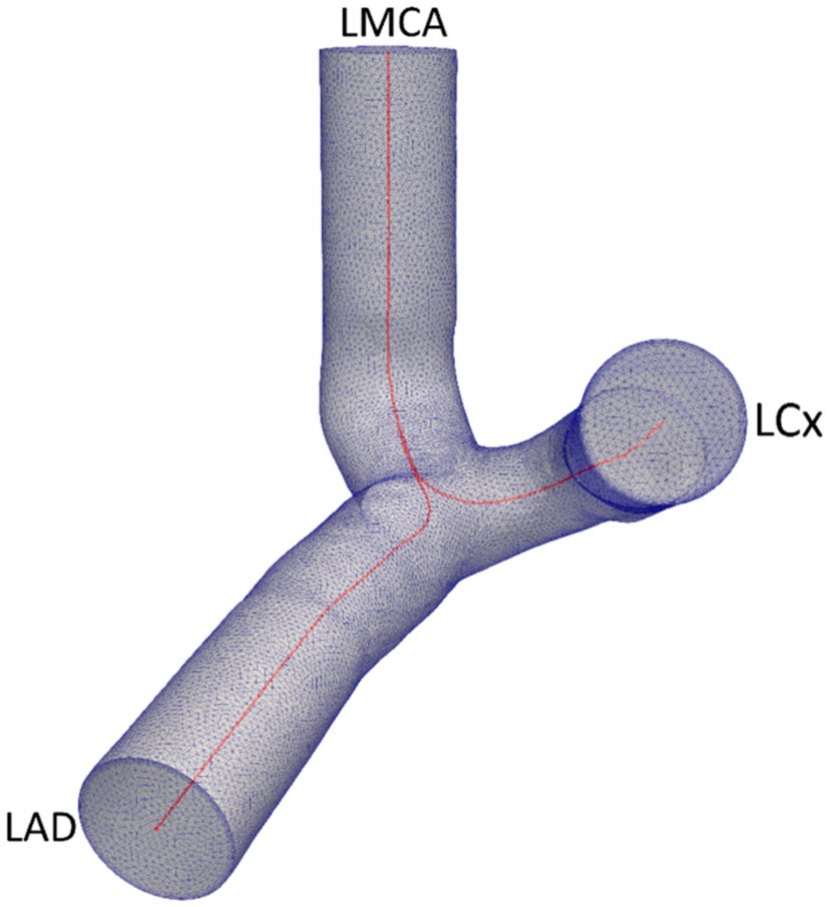
Computational dynamic mesh generated, with the vessel centerline shown in red.

We previously published on the process of data and image acquisition^36^. Briefly, coronary artery bifurcations were reconstructed from 127 (CTCA) images collected after obtaining written, informed consent with approval by the University of Auckland (Ref. 022,961) and UNSW Human Research Ethics Committees (Ref. HC190145). The research was conducted in accordance with the Declaration of Helsinki and the Australian National Statement on Ethical Conduct in Human Research. The patients had coronary artery disease symptoms and thus underwent a CTCA but showed no obstruction and had a zero-calcium score. For virtual reconstruction, the images were segmented using OsiriX 4.1.2 with the CMIV CTA plugin and the surface meshes were generated using MiaLite. Surface smoothing was carried out using the open-source Vascular Modelling Toolkit (version 1.4.0, VMTK) using Taubin’s Smoothing^37^ with a passband frequency of 0.03 and 30 iterations. An unstructured tetrahedral volumetric mesh was then created for computational analysis, which included entrance and exit extensions of four times the vessel radius. The mesh size and time-step were selected based on a sensitivity study, with < 1% change in TAWSS when doubling mesh density or halving timestep size (see supplementary material for details). A radius adaptive meshing method was used, that generates denser meshes in narrower regions, hence long or narrow vessels would have the highest number of elements. The average number of elements was 1.85 × 10^6^ ± 0.25 × 10^6^ (mean ± std. dev.) with an average density of 5790 ± 4373 elements/mm^3^ (std. dev. 4373). Five boundary layers were used to allow near wall values to be resolved accurately. Figure 2 shows an example mesh generated and the vessel centerline. The Vascular Modelling Toolkit (VMTK)^38^ was used to calculate and smooth the centerlines based on the surface mesh. Each branch of the bifurcation was sliced after a length of 10 mm from the bifurcation point. The centerlines were then resampled with equal spacing of 0.01 mm to minimize errors that would be introduced based on discretization.

### Tortuosity metrics

**Figure 3:**
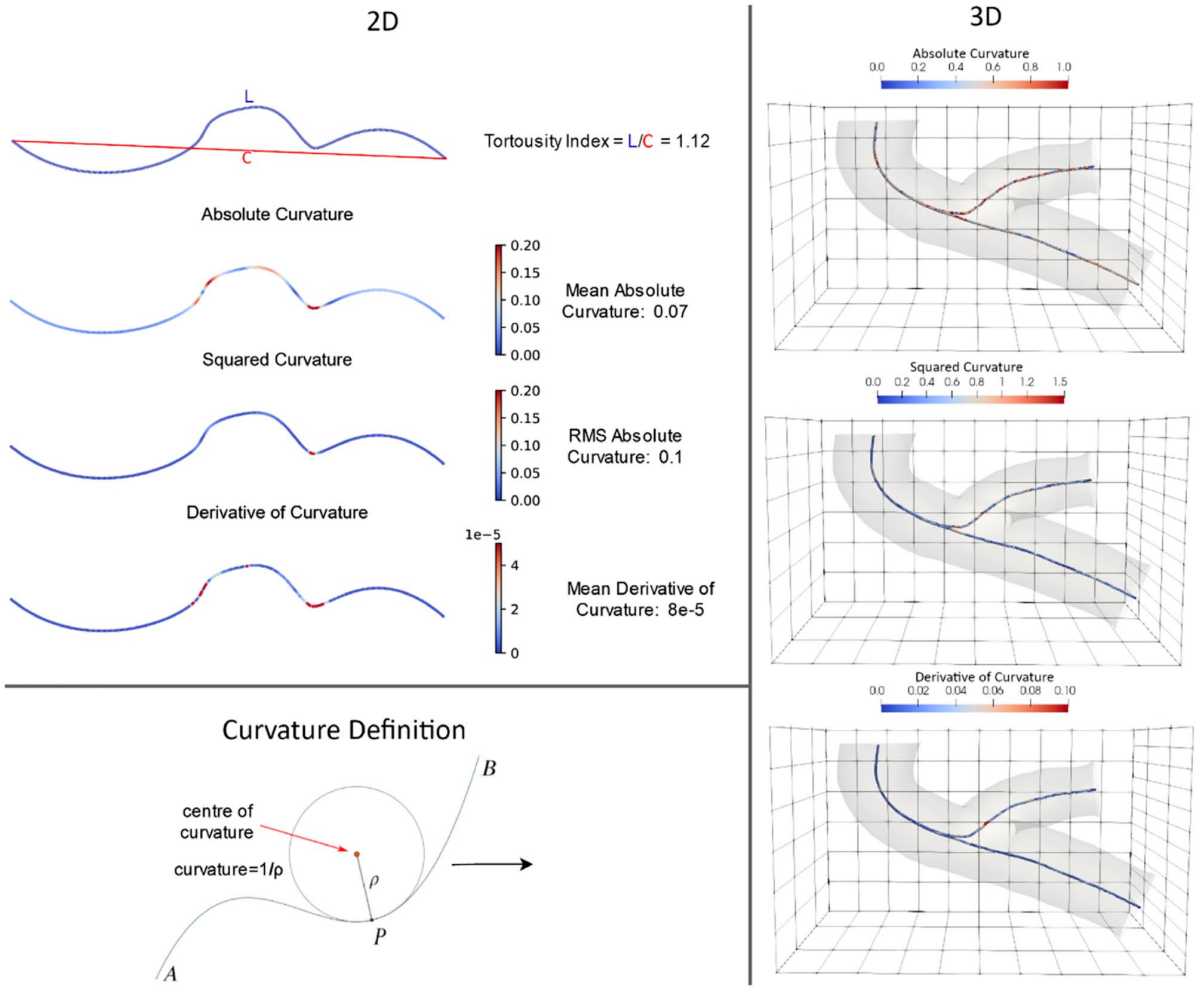
Sample representation of analyzed tortuosity metrics. Top left, the tortuosity index is the ratio of the length of the centerline (L) to the chord between its ends (C). The metrics used to calculate tortuosity index represented here through the centerline of the vessels because of curvature measures. The absolute-curvature is the normalized mean of the curvature moduli over the centerline, while the RMS curvature penalizes larger values of curvature. The average squared-derivative-curvature rises when there is a sudden change in curvature.

Major measures of tortuosity are 3-dimensional and include the tortuosity index, average absolute-curvature, RMS curvature, and average squared-derivative-curvature, which are explained in detail, as follows, and in Table 1 and Fig. 3.

**Table 1:** Overview of tortuosity measures.

Measure	Symbol	Formula	Previous work which used these tortuosity measures
Tortuosity index	${\varvec{\tau}}$	$\frac{{\varvec{L}}}{{\varvec{C}}}$	^1,2,30^
Total absolute-curvature*	${{\varvec{\kappa}}}_{{\varvec{t}}{\varvec{a}}}$	${\int }_{{{\varvec{t}}}_{1}}^{{{\varvec{t}}}_{2}}{\varvec{\kappa}}({\varvec{t}})\boldsymbol{ }{\varvec{d}}{\varvec{t}}$	^31,39,40^
Total squared-curvature*	${{\varvec{\kappa}}}_{tr}$	${\int }_{{{\varvec{t}}}_{1}}^{{{\varvec{t}}}_{2}}{{\varvec{\kappa}}}^{2}({\varvec{t}})\boldsymbol{ }{\varvec{d}}{\varvec{t}}$	^31,41^
Average absolute-curvature	${{\varvec{\kappa}}}_{{\varvec{a}}}$	$\frac{{\int }_{{{\varvec{t}}}_{1}}^{{{\varvec{t}}}_{2}}{\varvec{\kappa}}({\varvec{t}})\boldsymbol{ }{\varvec{d}}{\varvec{t}}}{L}$	^31^
RMS-curvature	${{\varvec{\kappa}}}_{r}$	$\sqrt{\frac{{\int }_{{{\varvec{t}}}_{1}}^{{{\varvec{t}}}_{2}}{{\varvec{\kappa}}}^{2}({\varvec{t}})\boldsymbol{ }{\varvec{d}}{\varvec{t}}}{L}}$	^31^
Average squared-derivative-curvature	${{\varvec{\kappa}}}_{{\varvec{d}}}$	$\frac{{\int }_{{{\varvec{t}}}_{1}}^{{{\varvec{t}}}_{2}}{(\frac{{\varvec{d}}{\varvec{\kappa}}({\varvec{t}})}{{\varvec{d}}{\varvec{t}}})}^{2}\boldsymbol{ }{\varvec{d}}{\varvec{t}}}{{\varvec{L}}}$	^35^

*L* = *length of vessel, C* = *length of chord between vessel ends,* $\kappa$ = *curvature.*

*The total absolute-curvature and the total squared curvature are not considered in this study, since these metrics are not scale invariant and dependent on the arc length of the vessels.

- **Tortuosity Index:** The most commonly used tortuosity measure, which is the ratio of the length of a vessel segment to the distance between its start and endpoint^1,2^**.**
- **Average Absolute-Curvature and RMS-Curvature:** Curvature, as considered here, is the reciprocal of the radius of curvature at a particular point on the centerline of the vessel. The Average Absolute-Curvature and RMS Curvature represent the absolute average and the root mean square over the physical length of the vessel respectively^31,34^.
- **Average Squared-Derivative-Curvature:** Equal to the integral of the square of the derivative of the curvature, averaged over vessel length; described by Patasius et al.^35^ as another curvature-based tortuosity measure.

The described tortuosity metrics were calculated separately for the LMCA, LAD and LCx vessels, yielding a total of 12 values (4 metrics × 3 branches) for every patient (× 127) forming a total of 1524 considerations.

### Computational modelling

A parabolic flow profile was prescribed at the inlet and constant pressure of 0 atm at the outlet^24^. The constant pressure outlet has also been a common assumption in published literature^42,43^ and is generally accepted for healthy vessels since realistic outlet conditions are often not available, and because of previous published validation with in vitro data^44^. The velocity–time profile and flow rate were adapted from previous studies^45^, and the flow was scaled according to equation developed by Giessen et al. ($q=1.43{d}^{1.55}$)^46^ using the inlet radii^47^. The Carreau–Yasuda model^48^ was used to simulate blood rheology. The walls of the artery were assumed to be rigid, in line with a previous study demonstrating that the modeling fluid–structure interaction led to less than 2% changes in TAWSS^49^. These boundary conditions follow the recommendations based on the published recommendations^50^ for cardiovascular studies. The CFD analyses were conducted using ANSYS CFX (version 18.2) on a high-performance computing cluster (Katana, University of New South Wales) using 16 core Intel Xeon CPUs and 32 GB RAM. Each simulation took an average of 2 h and involved an initial steady-state simulation followed by a transient simulation spanning four cardiac cycles. Results were obtained from the fourth cycle to minimize transient start-up effects. The High Resolution Scheme is used to discretize the advection terms of the Navier Stokes equations, and the Second Order Backward Euler scheme is used to discretize the transient terms. The CFL number was below 5^51^ and raw residuals of the governing equations are normalized^52^ to satisfy a convergence criterion of 10^−4^ for all variables. The average Reynolds Number was below 120, and a laminar fluid model was applied.

### Statistical analysis

The result parameter for the 127 patient-specific bifurcations was the normalized vessel area exposed to the commonly used threshold of TAWSS < 0.4 Pa, which is considered as non-physiologically low^53^. Only the original bifurcation region, without the flow extensions, was considered for the area calculation. A multivariate linear regression analysis was conducted to assess the performance of each tortuosity metric in explaining low shear stress. Models with the highest coefficient of determination (adjusted for number of predictors, i.e. adjusted R^2^) were deemed to be superior in predicting low shear stress prevalence, as they explain a larger portion of the shear stress variation. The obtained values of *p*-value were divided into three groups, indicating a significance level of 0.05 (*), 0.01 (**) and 0.001 (***).

## Results

### Tortuosity values

**Figure 4:**
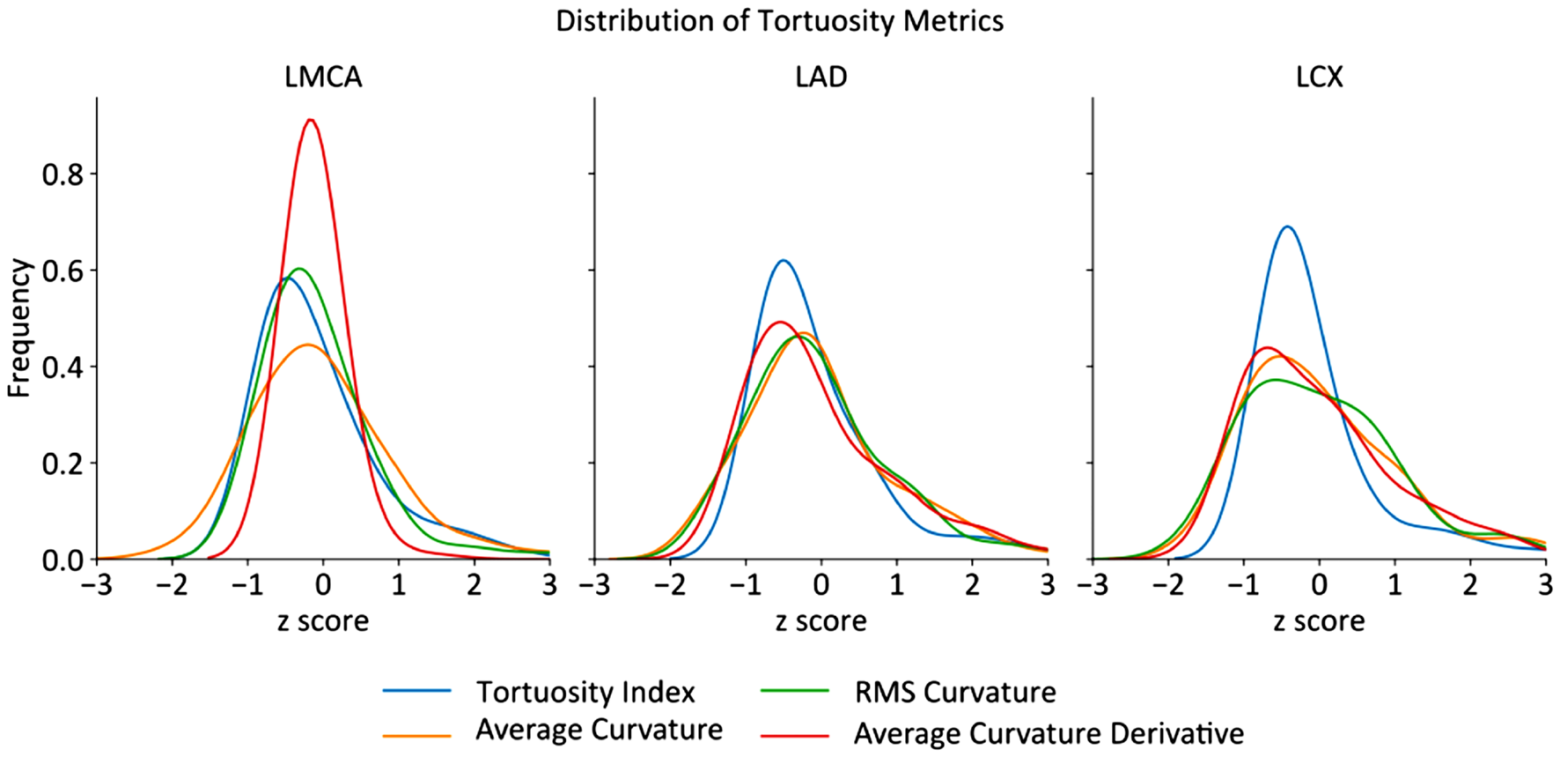
Normalized distribution of tortuosity metrics (n = 127).

Table 2 shows the mean and standard deviation of the tortuosity measures tested. As these measures have a very different mean and standard deviation, it would be prudent to first normalize the distributions to the same mean and standard deviation. Figure 4 shows the normalized distribution of the tortuosity metrics. Table 3 shows statistical measures of the distribution’s shape measuring kurtosis, how thick the distribution’s tails are compared to a normal distribution, which has a kurtosis of 0, and skewness, with zero skewness indicating a symmetrical distribution and positive skewness indicating right-skewed distribution. The tortuosity index shows large deviations from a normal distribution with large values of skewness and kurtosis. Average curvature, while still not statistically normally distributed, is generally the closest tortuosity metric to a normal distribution. This would reduce the spurious effects on common statistical methods with the assumption of the data being normally distributed.

**Table 2:** Mean ($\overline{x }$) and SD values of tortuosity metrics studied in 127 left main bifurcations.

Metric		LMCA		LAD		LCx	
		$\overline{x }$	± SD	$\overline{x }$	± SD	$\overline{x }$	± SD
Tortuosity index	$\tau$	1.01	0.01	1.02	0.03	1.03	0.03
Average absolute-curvature	${\kappa }_{a}$	0.40	0.11	0.51	0.10	0.50	0.08
RMS-curvature	${\kappa }_{r}$	0.67	0.17	0.76	0.14	0.74	0.11
Average squared-derivative curvature	${\kappa }_{d}$	0.06	0.03	0.08	0.03	0.07	0.02

**Table 3:** Kurtosis and skewness of tortuosity metrics studied in 127 left main bifurcations and LAD and LCx branches.

Metric	LMCA		LAD		LCX		Whole bifurcation	
	Kurtosis	Skew	Kurtosis	Skew	Kurtosis	Skew	Kurtosis	Skew
Tortuosity index	10.13	2.71	8.06	2.53	10.18	2.9	9.46	2.71
Average-curvature	2.38	1.12	1.43	1.02	0.64	0.91	1.49	1.02
RMS-curvature	20.65	3.76	1.99	1.21	0.21	0.67	7.62	1.88
Average squared-derivative-curvature	63.25	7.68	1.52	1.29	0.51	1.0	21.76	3.32

### Correlation to low TAWSS

**Figure 5:**
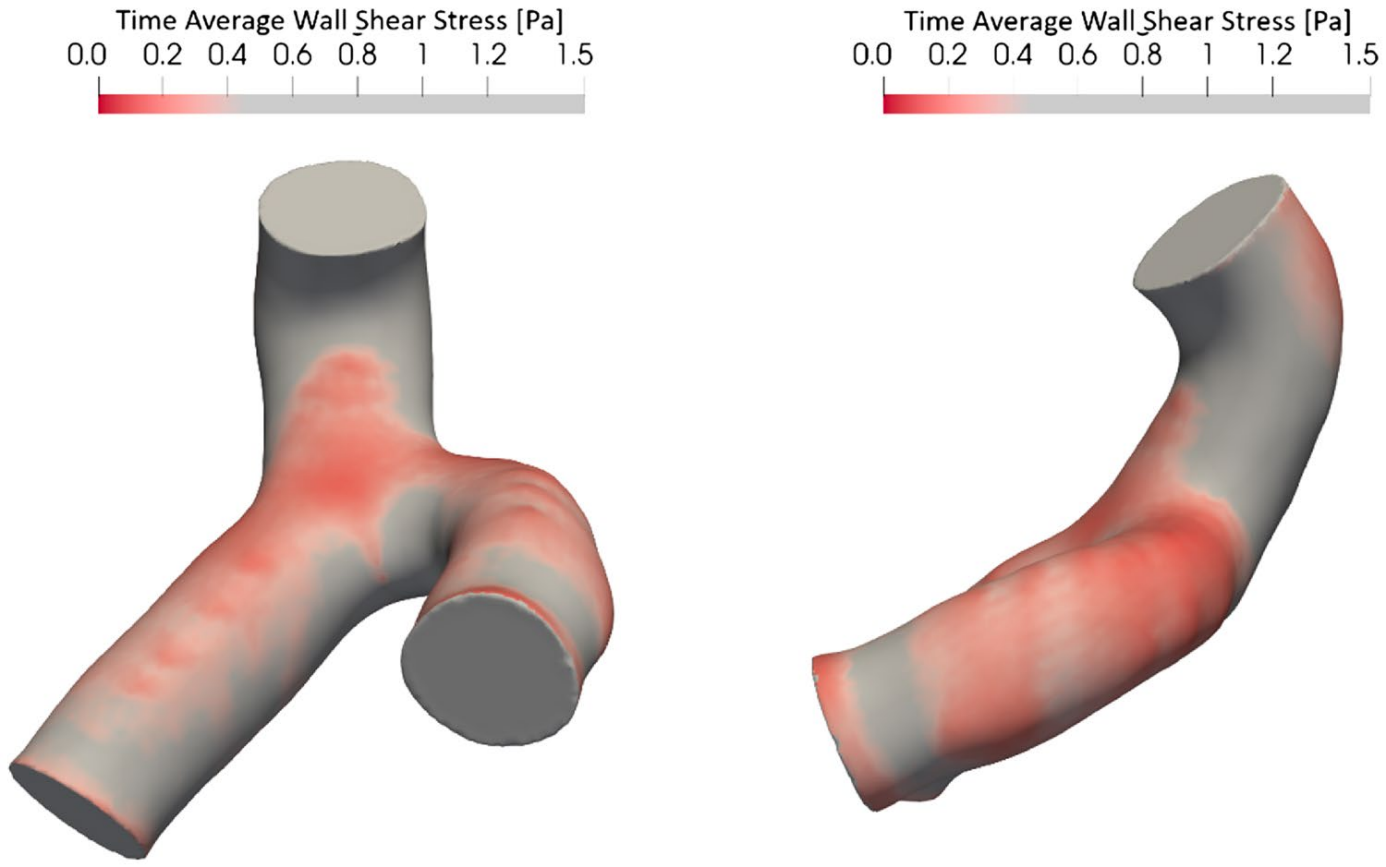
Example of low Time Average Wall Shear Stress (TAWSS) contour.

Results for all measures are summarized in Table 4, reporting R^2^ and *p*-values for all left main segments in all 127 cases. The absolute-curvature showed a significant correlation to the percentage vessel area coverage of low TAWSS in the LMCA and LAD segments, and a similar trend for the LCx. For both RMS curvature and average squared-derivative-curvature, a significant correlation is observed for the LMCA and LAD, yet there is no trend for the LCx segment. Interestingly, for the commonly used tortuosity index, no correlation is observed overall (*p* > 0.05). For the LCx branch, the only correlation is observed for the absolute-curvature, (*p* < 0.05), making this the only metric with a statistically significant correlation between low TAWSS and LCx value. A sample low TAWSS contour is shown in Fig. 5. It can be seen that vessels with higher curvature result in areas of low TAWSS on the outer wall of the curve.

**Table 4:** Tortuosity metrics statistical correlation to the low TAWSS < 0.4 Pa normalized vessel area coverage.

	LMCA		LAD		LCx		Complete bifurcation	
	R^2^	*p*-value	R^2^	*p*-value	R^2^	*p*-value	R^2^	*p*-value
Tortuosity index	− 0.011	0.660	0.026	0.099	0.007	0.276	− 0.018	0.865
Average absolute-curvature	0.196	< 0.001***	0.232	< 0.001***	0.061	0.013*	0.112	0.001***
RMS-curvature	0.113	< 0.001***	0.164	< 0.001***	0.010	0.236	0.093	0.002**
Average squared-derivative-curvature	0.086	0.003**	0.160	< 0.001***	0.007	0.274	0.101	0.001***

Higher R^2^ indicates better performance, i.e. the predictors account for a larger proportion of the variance observed in TAWSS. Note that R^2^ has been adjusted for multiple predictors and hence may be negative particularly for underperforming models.

*p ≤ 0.05; **p ≤ 0.01; ***p ≤ 0.001.

## Discussion

The current work utilized a statistical analysis of CFD results from the evaluation of 127 patient-specific left main coronary bifurcations, to determine which among four tortuosity metrics—tortuosity index, average absolute-curvature, RMS-curvature, or average squared-derivative-curvature—would better explain variations in TAWSS.

First, we demonstrated that curvature-based metrics consistently perform better than the tortuosity index—the most widely used metric in literature, explaining a significantly larger proportion of the variation in shear stress between patients. Curvature based metrics accounted for 6–27% of TAWSS variance, with an average of 10% for the whole bifurcation, compared to the tortuosity index, which could not explain any amount of TAWSS variance. Second, the tortuosity metrics show different distributions, with the average curvature metric closest to a normal distribution and the tortuosity index being the most dissimilar to normal distribution. Moreover, we did not find a significant relationship between the tortuosity index and low TAWSS. However, a significant positive relationship was discovered between curvature-based indices and low TAWSS area coverage.

Curvature-based metrics are based on the change of the vessel centerline trajectory from a straight path. These variations will require changes in blood flow direction and can have a notable impact on TAWSS. Compared to average absolute-curvature, RMS-curvature additionally accentuates sharp turns and average squared-derivative-curvature accentuates segments with rapidly changing curvature. Our results showed average absolute-curvature performing better than others, indicating the additional complexity accounted for by the other two metrics did not play a significant role.

Nevertheless, in clinical context, with coronary angiograms considered the ‘gold standard’ when assessing coronary artery pathology, only 2D representations of 3D coronary vessels are imaged. In CTCA studies, much of the vessel analysis is also performed on raw 2D images, because 3D reconstructions are considered more prone to error magnification. Therefore, the tortuosity index is currently of great popularity in the clinical setting. However, based on our findings, the average curvature-based metrics appears to be markedly superior to the tortuosity index in predicting the occurrence of a low TAWSS in coronary arteries.

Previous studies that have used tortuosity index to correlate shear stress in coronary arteries^2,13^ have obtained higher averages and wider spreads of TAWSS in comparison to our reported values here. However, these studies are not directly comparable as they measured tortuosity beyond the LMCA segment where tortuosity characteristics may change. Moreover, a large range of methodologies has previously been used within literature which hinders a clear comparison of results. For example, previous studies have calculated curvature immediately at the bifurcation point^13^ rather than for the whole vessel segment, combined LMCA-LAD and LMCA-LCX branches rather than considering them separately^2^, modeled a steady-state fluid flow instead of accounting for the transient cardiac cycle^2^, or used the total curvature metric instead of the average^2,13^, which is not scale-invariant and would be affected by the length of the vessels. Small sample sizes in previous literature (maximum of 8 patient cases^2,13^) may also contribute to disagreement in comparison to our 127 patient cases. To our knowledge, no other study has utilized RMS-curvature or average squared-derivative-curvature for coronary arteries before, yet these have been found relevant for retinal vasculature^31,35^.

A limitation of this work is that other coronary shape characteristics, such as the vessel diameter profile, may also affect the hemodynamic correlation predicted and may even have interdependent effects with tortuosity^54^. However, as this is a comparative analysis, it should be interpreted not as an attempt to predict TAWSS solely from tortuosity since other arterial geometric characteristics likely have an interdependent effect^55^, and thus it is sensible that the R^2^ values obtained are low. Rather, this work presents a comparison between different tortuosity metrics in a large coronary artery cohort. Near-wall effects and the surface roughness of arterial walls after reconstruction may also impact the quantitative values of hemodynamic computed in a similar manner^56^. Additionally, a constant pressure outlet condition is used due to the unavailability of patient-specific data, which may result in different behavior compared to in-vivo blood flow. Finally, this work only considers the left main bifurcation, and the results may not be applicable to downstream vessels.

Overall, previous investigators used different tortuosity metrics that make uniform interpretations of results across studies difficult. Here, we showcased the variations in the correlations with TAWSS as a result of these metrics, highlighting the need for a predictive tortuosity metric. We demonstrated that curvature-based metrics perform better than the commonly used tortuosity index when correlating to coronary bifurcation hemodynamics, possibly since they account for small-scale anatomical variations within the vessel and are independent of the vessel distance considered.

With the tortuosity index not accurately capturing a “global” metric of a curve, this result is overall not surprising. However, its prominence to date in research is largely derived from the ease of use and applicability to common 2D imaging in the clinical context. Still, we hope to herewith increase the understanding of such assessment to advocate its significant shortcomings for meaningful results, which may have mitigated coherency in interpretation of relevant clinical observations to date. Hence, we propose that the average absolute-curvature is a more appropriate metric for coronary tortuosity considerations rather than the commonly used tortuosity index and should therefore be used as a new benchmark measure in future research. Clinically, this may help to better link coronary artery anatomical anomalies to adverse clinical events such as SCAD.

## Supplementary Information

Supplementary Information.

## Data Availability

The datasets generated and analyzed during the current study are available from the corresponding author on reasonable request.

## Abbreviations

CFD: Computational fluid dynamics
CTCA: Computed tomography coronary angiography
LMCA: Left main coronary artery
LAD: Left anterior descending artery
LCx: Left circumflex artery
RMS: Root-mean-squared
SCAD: Spontaneous coronary artery dissection
TAWSS: Time average wall shear stress
$$d$$: Vessel diameter
$q$: Volumetric flow rate
$$\tau$$: Tortuosity index
${\kappa }_{a}$: Average-absolute-curvature
$${\kappa }_{r}$$: RMS-curvature
${\kappa }_{d}$: Average-squared-derivative-curvature

## References

[1] Han H (2012). Twisted blood vessels: Symptoms, etiology and biomechanical mechanisms. J. Vasc. Res..

[2] Malvè M, Gharib A, Yazdani S, Finet G, Martínez M, Pettigrew R, Ohayon J (2015). Tortuosity of coronary bifurcation as a potential local risk factor for atherosclerosis: CFD steady state study based on in vivo dynamic CT measurements. Ann. Biomed. Eng..

[3] Vorobtsova N, Chiastra C, Stremler M, Sane D, Migliavacca F, Vlachos P (2016). Effects of vessel tortuosity on coronary hemodynamics: An idealized and patient-specific computational study. Ann. Biomed. Eng..

[4] Eleid M, Guddeti R, Tweet M, Lerman A, Singh M, Best P, Vrtiska T, Prasad M, Rihal C, Hayes S, Gulati R (2014). Coronary artery tortuosity in spontaneous coronary artery dissection: Angiographic characteristics and clinical implications. Circ. Cardiovasc. Interven..

[5] Ciurică S, Lopez-Sublet M, Loeys B, Radhouani I, Natarajan N, Vikkula M, Maas A, Adlam D, Persu A (2019). Arterial tortuosity: Novel implications for an old phenotype. Hypertension.

[6] Hiroki M, Miyashita K, Oda M (2002). Tortuosity of the white matter medullary arterioles is related to the severity of hypertension. Cerebrovasc. Dis..

[7] Buradi A, Mahalingam A (2020). Impact of coronary tortuosity on the artery hemodynamics. Biocybern. Biomed. Eng..

[8] Caro C, Fitz-Gerald J, Schroter R (1971). Atheroma and arterial wall shear-observation, correlation and proposal of a shear dependent mass transfer mechanism for atherogenesis. Proc. R. Soc. Lond. Ser. B Biol. Sci..

[9] Chiha J, Mitchell P, Gopinath B, Burlutsky G, Kovoor P, Thiagalingam A (2017). Gender differences in the prevalence of coronary artery tortuosity and its association with coronary artery disease. IJC Heart Vasc..

[10] Kahe F, Sharfaei S, Pitliya A, Jafarizade M, Seifirad S, Habibi S, Chi G (2020). Coronary artery tortuosity: A narrative review. Coron. Artery Dis..

[11] Li Y, Shen C, Ji Y, Feng Y, Ma G, Liu N (2011). Clinical implication of coronary tortuosity in patients with coronary artery disease. PLoS ONE.

[12] Khosravani-Rudpishi M, Joharimoghadam A, Rayzan E (2018). The significant coronary tortuosity and atherosclerotic coronary artery disease; What is the relation?. J. Cardiovasc. Thorac. Res..

[13] Pinho N, Castro C, António C, Bettencourt N, Sousa L, Pinto S (2019). Correlation between geometric parameters of the left coronary artery and hemodynamic descriptors of atherosclerosis: FSI and statistical study. Med. Biol. Eng. Comput..

[14] Weydahl E, Moore J (2001). Dynamic curvature strongly affects wall shear rates in a coronary artery bifurcation model. J. Biomech..

[15] Liu G, Wu J, Ghista D, Huang W, Wong K (2015). Hemodynamic characterization of transient blood flow in right coronary arteries with varying curvature and side-branch bifurcation angles. Comput. Biol. Med..

[16] Pinho N, Sousa L, Castro C, António C, Carvalho M, Ferreira W, Ladeiras-Lopes R, Ferreira N, Braga P, Bettencourt N, Pinto S (2019). The impact of the right coronary artery geometric parameters on hemodynamic performance. Cardiovasc. Eng. Technol..

[17] Nafia, T., Handayani, A. & Mengko, T. Evaluation of retinal vascular tortuosity indexes. In *5th International Conference on Instrumentation, Communications, Information Technology, and Biomedical Engineering (ICICI-BME)*, 111–116 (2017).

[18] Schilt S, Moore J, Delfino A, Meister J (1996). The effects of time-varying curvature on velocity profiles in a model of the coronary arteries. J. Biomech..

[19] Kirpalani A, Park H, Butany J, Johnston K, Ojha M (1999). Velocity and wall shear stress patterns in the human right coronary artery. J. Biomech. Eng..

[20] Iwami T, Fujii T, Miura T, Otani N, Iida H, Kawamura A, Yoshitake S, Kohno M, Hisamatsu Y, Iwamoto H, Matsuzaki M (1998). Importance of left anterior descending coronary artery curvature in determining cross-sectional plaque distribution assessed by intravascular ultrasound. Am. J. Cardiol..

[21] Stone P, Coskun A, Kinlay S, Clark M, Sonka M, Wahle A, Ilegbusi O, Yeghiazarians Y, Popma J, Orav J (2003). Effect of endothelial shear stress on the progression of coronary artery disease, vascular remodeling, and in-stent restenosis in humans: In vivo 6-month follow-up study. Circulation.

[22] LaDisa J, Olson L, Molthen R, Hettrick D, Pratt P, Hardel M, Kersten J, Warltier D, Pagel P (2005). Alterations in wall shear stress predict sites of neointimal hyperplasia after stent implantation in rabbit iliac arteries. Am. J. Physiol.-Heart Circ. Physiol..

[23] Morlacchi S, Keller B, Arcangeli P, Balzan M, Migliavacca F, Dubini G, Gunn J, Arnold N, Narracott A, Evans D (2011). Hemodynamics and in-stent restenosis: Micro-CT images, histology, and computer simulations. Ann. Biomed. Eng..

[24] Chaichana T, Sun Z, Jewkes J (2013). Haemodynamic analysis of the effect of different types of plaques in the left coronary artery. Comput. Med. Imaging Graph..

[25] Kreyszig E (1991). Differential Geometry.

[26] Lee S, Antiga L, Spence J, Steinman D (2008). Geometry of the carotid bifurcation predicts its exposure to disturbed flow. Stroke.

[27] Kim B, Kim S, Kang D, Kwon S, Suh D, Kim J (2015). Vascular tortuosity may be related to intracranial artery atherosclerosis. Int. J. Stroke.

[28] Lee J, Jang L, Sun W, Park J, Choi J (2011). Relationship between tortuosity and atherosclerotic changes of the abdominal aorta. Korean J. Vasc. Endovasc. Surg..

[29] Gallo D, Steinman D, Morbiducci U (2015). An insight into the mechanistic role of the common carotid artery on the hemodynamics at the carotid bifurcation. Ann. Biomed. Eng..

[30] Smedby O, Högman N, Nilsson S, Erikson U, Olsson A, Walldius G (1993). Two-dimensional tortuosity of the superficial femoral artery in early atherosclerosis. J. Vasc. Res..

[31] Hart, W., Goldbaum, M., Côté, B., Kube, P. & Nelson, M. Automated measurement of retinal vascular toruosity. In* Proceedings on AMIA Annual Fall Symposium* (1997). PMC2233372 9357668

[32] Hart W, Goldbaum M, Côté B, Kube P, Nelson M (1999). Measurement and classification of retinal vascular tortuosity. Int. J. Med. Inform..

[33] Nafia, T. & Handayani, A. Quantification of retinal vascular tortuosity: Evaluation on different numbers of sampling points. In* 2nd International Conference on Biomedical Engineering (IBIOMED)*, 39–43 (2018).

[34] Cheung C, Tay W, Mitchell P, Wang J, Hsu W, Lee M, Lau Q, Zhu A, Klein R, Saw S, Wong T (2011). Quantitative and qualitative retinal microvascular characteristics and blood pressure. J. Hypertens..

[35] Patasius, M., Marozas, V., Lukosevicius, A. & Jegelevicius, D. Evaluation of tortuosity of eye blood vessels using the integral of square of derivative of curvature. In* EMBEC'05: Proceedings on 3rd IFMBE European Medical and Biological Engineering Conference*, 1589 (2005).

[36] Medrano-Gracia P, Ormiston J, Webster M, Young A, Beier S, Ellis C, Wang C, Smedby Ö, Cowan B (2016). A computational atlas of normal coronary artery anatomy. EuroIntervention.

[37] Taubin, G. Curve and surface smoothing without shrinkage. In* Proceedings on IEEE international Conference on Computer Vision*, 852–857 (1995).

[38] Vascular Modeling Toolkit. http://www.vmtk.org/. Accessed 22 September 2021.

[39] Bhuiyan A, Nath B, Ramamohanarao K, Kawasaki R, Wong T (2010). Automated analysis of retinal vascular tortuosity on color retinal images. J. Med. Syst..

[40] O’Flynn P, O’Sullivan G, Pandit A (2007). Methods for three-dimensional geometric characterization of the arterial vasculature. Ann. Biomed. Eng..

[41] Dougherty G, Johnson M, Wiers M (2010). Measurement of retinal vascular tortuosity and its application to retinal pathologies. Med. Biol. Eng. Comput..

[42] Mohammadi H, Bahramian F (2009). Boundary conditions in simulation of stenosed coronary arteries. Cardiovasc. Eng..

[43] Boutsianis E, Dave H, Frauenfelder T, Poulikakos D, Wildermuth S, Turina M, Ventikos Y, Zund G (2004). Computational simulation of intracoronary flow based on real coronary geometry. Eur. J. Cardio-Thorac. Surg..

[44] Beier S, Ormiston J, Webster M, Carter J, Norris S, Medrano-Garcia P, Young A, Cowan B (2016). Dynamically scaled phantom phase contrast MRI compared to true-scale computational modeling of coronary artery flow. J. Magn. Reson. Imaging.

[45] Nichols W, O'Rourke M, Vlachopoulos C, Hoeks A, Reneman R (2011). The Coronary Circulation in McDonald's Blood Flow in Arteries: Theoretical, Experimental and Clinical Principles.

[46] van der Giessen A, Groen H, Doroit P, de Feyter P, van der Steen A, van de Vosse F, Wentzel J, Gijsen F (2011). The influence of boundary conditions on wall shear stress distribution in patients specific coronary trees. J. Biomech..

[47] Formaggia L, Lamponi D, Quarteroni A (2003). One-dimensional models for blood flow in arteries. J. Eng. Math..

[48] Johnston B, Johnston P, Corney S, Kilpatrick D (2006). Non-Newtonian blood flow in human right coronary arteries: Transient simulations. J. Biomech..

[49] Chiastra C, Migliavacca F, Martinez M, Malve M (2014). On the necessity of modelling fluid–structure interaction for stented coronary arteries. J. Mech. Behav. Biomed. Mater..

[50] Gijsen F, Katagiri Y, Barlis P, Bourantas C, Collet C, Coskun U, Daemen J, Dijkstra J, Edelman E, Evans P, van der Heiden K, Hose R, Koo B, Krams R, Marsden A, Migliavacca F, Onuma Y, Ooi A, Poon E, Samady H, Stone P, Takahashi K, Tang D, Thondapu V, Tenekecioglu E, Timmins L, Torii R, Wentzel J, Serruys P (2019). Expert recommendations on the assessment of wall shear stress in human coronary arteries: Existing methodologies, technical considerations, and clinical applications. Eur. Heart J..

[51] Norris S (2000). A Parallel Navier Stokes Solver for Natural Convection and Free Surface Flow.

[52] Ansys I (2021). ANSYS CFX-Solver Theory Guide.

[53] Malek A, Alper S, Izumo S (1999). Hemodynamic shear stress and its role in atherosclerosis. JAMA.

[54] Beier S, Ormiston J, Webster M, Cater J, Norris S, Medrano-Gracia P, Young A, Cowan B (2017). Vascular Hemodynamics with Computational Modeling and Experimental Studies. Computing and Visualization for Intravascular Imaging and Computer-Assisted Stenting.

[55] Beier S, Ormiston J, Webster M, John C, Norris S, Medrano-Gracia P, Young A, Conan B (2016). Impact of bifurcation angle and other anatomical characteristics on blood flow—A computational study of non-stented and stented coronary arteries. J. Biomech..

[56] Owen D, Schenkel T, Shepherd D, Espino D (2020). Assessment of surface roughness and blood rheology on local coronary haemodynamics: A multi-scale computational fluid dynamics study. J. R. Soc. Interface.

