# Supplementary material for: Accuracy of vascular tortuosity measures using computational modelling
Source: Sci Rep. 2022 Jan 17;12:865. doi: 10.1038/s41598-022-04796-w (PMC8764056; doi:10.1038/s41598-022-04796-w)
Supplement: Supplementary file 1 — Supplementary Information. [file 41598_2022_4796_MOESM1_ESM.docx]

**Accuracy of vascular tortuosity measures using computational modelling**

Vishesh Kashyap^a†^, Ramtin Gharleghi^b†*^, Darson D. Li^b^, Lucy McGrath-Cadell^c^,

Robert M. Graham^c^, Chris Ellis ^d^, Mark Webster^e^, Susann Beier^b^

^a^ Mechanical and Aerospace Engineering Department, Henry Samueli School of Engineering and Applied Science, University of California, Los Angeles, United States

^b^ School of Mechanical and Manufacturing Engineering, University of New South Wales, Sydney, New South Wales, Australia

^c^ Molecular Cardiology and Biophysics Division, Victor Chang Cardiac Research Institute, Sydney, New South Wales, Australia

^d^ Auckland Heart Group, Auckland, New Zealand

^e^ Auckland City Hospital, Auckland, New Zealand

# **Timestep sensitivity**

|  | Percentage difference when halving timestep size | |
| --- | --- | --- |
| Time step size [s] | WSS @Peak Flow rate | TAWSS |
| 0.002 | 2.87% | 0.84% |
| 0.004 | 14.40% | 0.84% |
| 0.006 | 14.26% | 0.85% |
| 0.008 | 13.39% | 0.85% |

1. **Mesh Sensitivity**

|  | Elements | | Low TAWSS Area | |  |
| --- | --- | --- | --- | --- | --- |
| Case | Current Mesh | 2x Dense Mesh | Current Mesh | 2x Dense Mesh | % Difference |
| 1 | 1943474 | 4050389 | 76.3% | 76.8% | 0.6% |
| 2 | 1906815 | 3946383 | 64.1% | 64.1% | 0.0% |
| 3 | 1976460 | 4018546 | 65.3% | 65.9% | 0.9% |
| 4 | 1813074 | 3764759 | 78.5% | 79.0% | 0.6% |
| 5 | 1858774 | 3795326 | 57.2% | 57.8% | 1.0% |

1. **Segmentation**

OsiriX was used to semiautomatically extract vessel centerlines by marking the start and end points of the vessels within OsiriX. We then use Mia Lite with parameters 100, 950, 0.1 to generate segmentations of the vessel lumen.
